# Salivary Inflammatory Markers in Trichotillomania: A Pilot Study

**DOI:** 10.1159/000489865

**Published:** 2018-06-19

**Authors:** Jon E. Grant, Samuel R. Chamberlain

**Affiliations:** ^a^Department of Psychiatry and Behavioral Neuroscience, University of Chicago, Chicago, Illinois, USA; ^b^Department of Psychiatry, University of Cambridge, Cambridge and Peterborough NHS Foundation Trust, Cambridge, United Kingdom

**Keywords:** Trichotillomania, Inflammation, Interleukin-6, Interleukin-8, Interleukin-1beta, Tumor necrosis factor-alpha, Cytokines

## Abstract

**Background:**

Immune dysregulation has been hypothesized to be important in the development and pathophysiology of compulsive disorders such as obsessive compulsive disorder (OCD), which has a high comorbid overlap with trichotillomania (both are OC-related disorders). The role of inflammation in the pathophysiology of trichotillomania has garnered little research to date.

**Methods:**

Individuals with trichotillomania provided saliva sample for analysis of inflammatory cytokines. Additionally, these participants were examined on a variety of demographic variables (including body mass index [BMI], previously found to relate to inflammation) along with clinical measures (symptom severity, functioning, and comorbidity).

**Results:**

Thirty-one participants, mean age of 24.7 (±10.2) years, 27 (87.1%) females were ­included. The mean score on the Massachusetts General Hospital Hair Pulling Scale was 15.7 (±4.2), reflective of moderate symptom severity. Compared to normative data, the mean inflammatory marker levels in the trichotillomania sample had the following Z scores: interleukin-1β (IL-1β) *Z* = −0.26, IL-6 *Z* = −0.39, IL-8 *Z* = −0.32, and tumor necrosis factor-α *Z* = −0.83. Levels of inflammatory markers did not correlate significantly with BMI, depressive mood, symptom severity, or disability.

**Conclusions:**

The relatively low level of inflammatory saliva cytokines observed in the current study (negative z scores versus normative data with medium effect sizes) indicates that evaluation of blood inflammatory levels in trichotillomania versus matched controls would be valuable in future work. If a hypoinflammatory state is confirmed ­using blood samples, this would differentiate trichotillomania from other mental disorders (such as OCD, schizophrenia, and depression), which have typically been linked with high inflammatory measures in the literature, at least in some cases.

## Introduction

Trichotillomania is an under-recognized and oftentimes functionally impairing condition in which individuals repeatedly pull out their hair [1]. Although described in the medical literature for almost 2 centuries, trichotillomania remains poorly understood with limited data regarding its pathophysiology [2]. Trichotillomania constitutes a relatively specific type of behavior, characterized in terms of excessive grooming, which also occurs in other animal species. As such, across psychiatric disorders, the biological substrates of trichotillomania may be particularly well suited to translational modeling [3]. Obsessive compulsive disorder (OCD) and trichotillomania are both classified as Obsessive-Compulsive Related Disorders in the Diagnostic and Statistical Manual Version 5, in recognition of their high comorbidity, phenomenology (repetitive habitual behaviors), and possible overlapping involvement of dysfunctional fronto-striatal circuitry (striatum and frontal cortices) [2, 3].

There are several lines of evidence to suggest a role for immune-mediated pathophysiology in a variety of psychiatric conditions, including OCD, a disorder with phenomenological and possibly biological links to trichotillomania [4]. Studies measuring plasma cytokine levels have demonstrated a significant reduction in interleukin-1β (IL-1β) levels in OCD subjects compared with controls, but no significant differences in plasma levels of IL-6 and tumor necrosis factor-α (TNF-α) have been shown [5]. A recent case-control study of 20 adults with OCD who underwent PET imaging, however, found that translocator protein distribution volume (translocator protein density increases when microglia are activated during neuroinflammation and the distribution volume is an index of translocator protein density) was significantly elevated in the dorsal caudate, orbitofrontal cortex, thalamus, ventral striatum, and dorsal putamen [6] demonstrating inflammation within the neurocircuitry of OCD. The only study which included subjects with trichotillomania examined cerebrospinal fluid and found that the mean cerebrospinal fluid IL-6 levels did not differ between OCD patients (*n* = 26) and controls or between trichotillomania patients (*n* = 9) and their matched controls [7].

Cytokines are small, soluble proteins secreted by cells to influence the behavior of other cells via regulation of cellular immunity and inflammatory response. Pro-inflammatory cytokines include IL-1β, IL-6, and TNF-α, which are produced both peripherally and in the central nervous system. TNF-α stimulates vagal afferents and is produced by neurons and glial cells in an activity-dependent manner [8, 9]. IL-6 and IL-1 are also produced in the brain, where they mediate neuroinflammation and response to injury [9, 10]. Interestingly, IL-1β, IL-6, and TNF-α also mediate neuroprotection to excitotoxicity [11]. IL-8 is associated with inflammation and is increased by oxidant stress, which in a vicious cycle causes the recruitment of inflammatory cells and induces an increase in oxidant stress mediators. While poorly understood, it is increasingly recognized that there is important cross-talk between the peripheral immune system and central nervous system functioning, whereby inflammation may influence psychiatric symptoms and cognition [12, 13].

Although some studies shed some light on the topic of possible role inflammation in compulsive disorders, many questions remain unanswered. Understanding the role of cytokines in compulsive behaviors such as trichotillomania may allow for a greater understanding of the pathophysiology underlying this disorder and related disorders. Based on the extant literature, we hypothesized that salivary cytokine levels would be abnormal in trichotillomania. By examining the relationship between salivary cytokines and trichotillomania, we hope to determine a possible biological signal supportive of follow-up work using blood samples (which may more accurately capture central inflammatory pathways than saliva).

## Methods

### Ethical Approval

Study procedures were carried out in accordance with the Declaration of Helsinki. The Institutional Review Board at the University of Chicago approved the study and subjects provided written informed consent. In the case of non-adults, parental consent was obtained.

### Subjects

Data from 31 female participants with primary trichotillomania were included in this study. All subjects had a current Diagnostic and Statistical Manual Version 5 primary diagnosis of trichotillomania (American Psychiatric Association, 2013). Other inclusion criteria included age 10–66 years, the ability to be interviewed in person, and willingness to provide a saliva sample. Exclusion criteria included prior/current diagnosis of bipolar disorder or psychosis or current substance use disorder.

### Assessments

Individuals with a primary diagnosis of trichotillomania were examined using a semi-structured interview focusing on the clinical features of trichotillomania. Each participant (or their parent in the case of non-adults) provided their history of psychiatric disorders.

Body mass index (BMI) was calculated for each participant using the standard formula BMI = *w*/*h* squared, where *w* is the weight in kilograms and *h* is the height in meters. For the present analyses, obesity was defined as a BMI greater than 30 kg/m^2^, overweight as a BMI less than 30 kg/m^2^, but greater than or equal to 25 kg/m^2^, and normal weight as a BMI greater than 18.5 kg/m^2^ but less than 25 kg/m^2^. We recorded BMI because previous studies have found strong relationships between elevated BMI and inflammation in the context of depression and healthy controls [14].

Participants also provided a saliva sample via passive drool. Participants were not allowed to eat, drink, smoke, or use any oral hygiene products for at least 1 h before the saliva collection. Saliva levels of IL-1β, IL-6, IL-8, and TNF-α were quantified (for assay methodology, see www.salimetrics.com). We opted for saliva samples because they are minimally invasive and convenient, compared to blood tests. While saliva samples have relatively weak correlations with blood levels of cytokines (the strongest evidence is for a significant albeit relatively small correlation with IL-6) [15, 16], we believed that an initial pilot study using saliva would constitute a valuable research step; since significant associations between saliva inflammatory markers and trichotillomania would support future work using blood tests.

Severity of trichotillomania, depressive symptoms, psychosocial dysfunction, and impulsivity were assessed using the following measures:

The Massachusetts General Hospital Hair Pulling Scale (MGH-HPS) [17, 18]. The MGH-HPS is a valid and reliable 7-item, self-report scale that rates urges to pull hair, actual amount of pulling, perceived control over behavior, and distress associated with hair pulling over the preceding 7 days.

Mood and Feelings Questionnaire. Mood and Feelings Questionnaire 13-item adult short version, is a well validated instrument consisting of a series of descriptive phrases regarding how the subject has been feeling or acting most of the time, sometimes, or not at all during the previous 2 weeks [19].

Sheehan Disability Scale (SDS) [20]. The SDS is a valid and reliable scale that evaluates psychosocial dysfunction in 3 domains: work/school, social life, and home/family life.

Barratt Impulsiveness Scale, Version 11. The Barratt Impulsiveness Scale, Version 11 is a 30-item measure designed to assess impulsivity across 3 dimensions: attentional (inability to concentrate), motor (acting without thinking), and non-planning (lack of future orientation) [21].

### Data Analysis

Demographic, clinical, and salivary cytokine level data were presented in summary form. Relationships between demographic/clinical measures (age, education level, MGH-HPS, SDS total scores) and salivary cytokine (IL-1β, IL-6, IL-8, and TNF-α) were explored using correlation tests (Spearman's *r*; or Wilcoxon's *z* for gender comparisons). Statistical significance was defined as *p* < 0.05 2-tailed. We did not adjust the alpha level to reflect all statistical comparisons because this is one of the first studies of this topic and is therefore exploratory.

## Results

The sample comprised 31 individuals with trichotillomania (mean age of 24.7 ± SD 10.2 years), of whom most (27 [87.1%] were females. The mean total score on the MGH-HPS was 15.7 [± SD 4.2]), reflective of moderate trichotillomania symptom severity. Of the 31 participants, 10 (32.2%) had co-occurring skin picking disorder, but in each case, the trichotillomania was self-reported as their primary difficulty based on interference in their lives. Four (12.9%) had lifetime histories of major depressive disorder, with 9 (29.0%) taking antidepressants. Participants did not report any ongoing health issues and were not taking any other medications or supplements on a regular basis, other than 2 participants who reported occasional (i.e., less than 1 time per week) use of NSAIDS.

Saliva testing showed the following levels (all expressed in pg/mL): IL-1β (86.4 ± 103.2), IL-6 (3.6 ± 3.1), IL-8 (638.7 ± 622.7), and TNF-α (2.54 ± 1.98). Compared to normative data [16, 22], the mean inflammatory marker levels in the trichotillomania sample had the following *Z* scores: IL-1β *Z* = −0.26, IL-6 *Z* = −0.39, IL-8 *Z* = −0.32, and TNF-α *Z* = −0.83.

Salivary inflammatory levels correlated significantly with age (IL-1β *r* = 0.433, *p* = 0.015; IL-6 *r* = 0.361, *p* = 0.046; IL-8 *r* = 0.437, *p* = 0.014; TNF-α *r* = 0.375, *p* = 0.038). Inflammatory levels did not differ as a function of gender (IL-1β Wilcoxon *Z* = −0.56, *p* = 0.586; IL-6 *Z* = 1.03, *p* = 0.302; IL-8 *Z* < 0.01, *p* > 0.99; TNF-α *Z* = 0.147, *p* = 0.883). Inflammatory measures did not correlate with trichotillomania symptom severity (IL-1β *r* = −0.163, *p* = 0.407; IL-6 *r* < 0.01, *p* > 0.99; IL-8 *r* = 0.153, *p* = 0.558; TNF-α *r* = 0.080, *p* = 0.686), or with Sheehan disability (IL-1β *r* = −0.154, *p* = 0.433; IL-6 *r* < 0.01, *p* > 0.99; IL-8 *r* < 0.001, *p* > 0.99; TNF-α *r* = −0.061, *p* = 0.757), or Barratt scores (IL-1β *r* = −0.089, *p* = 0.735; IL-6 *r* = 0.325, *p* = 0.204; IL-8 *r* = 0.153, *p* = 0.558; TNF-α *r* = 0.130, *p* = 0.620), or BMI (IL-1β *r* = 0.309, *p* = 0.173; IL-6 *r* = 0.325, *p* = 0.204; IL-8 *r* = 0.153, *p* = 0.558; TNF-α *r* = 0.239, *p* = 0.300), or depressed mood (IL-1β *r* = 0.110, *p* = 0.655; IL-6 *r* = 0.319, *p* = 0.184; IL-8 *r* = 0.299, *p* = 0.214; TNF-α *r* = 0.063, *p* = 0.800). There were no differences between those participants taking or not taking an antidepressant.

## Discussion

Inflammatory dysfunction has been implicated in other mental health disorders such as OCD and schizophrenia [4, 12], but has received scant study in the context of trichotillomania. The current study examined a range of salivary inflammatory markers (IL-1β, IL-6, IL-8, and TNF-α) in a sample of patients with trichotillomania, and whether inflammation related to symptom severity and other clinical measures. The key finding was that patients overall had relatively low level of salivary inflammatory markers compared to externally published norms. Reduced autonomic response to painful stimulation, measured using the cold pressor task, has previously been reported in trichotillomania [23] and in skin picking disorder [24]. Many patients with trichotillomania report that hair pulling is not painful, especially after the habit has developed [25]. In the general population, cold pressor task pain perception was positively correlated with higher inflammation as indexed by C-reactive protein [26]. Acute inflammation in healthy volunteers, such as induced by an endotoxin model, increases pain response on the Cold Pressor test [27]. Therefore, 1 intriguing possibility raised by the present data is that inflammatory pathways are dampened in trichotillomania; and that this in turn may contribute to persistence of hair pulling due to reduced pain response. However, confirmation of this would require follow-up work using blood samples, which are more directly related to systemic inflammatory pathways, than saliva samples. In a prior meta-analysis, a significant reduction in IL-1β levels was found in OCD subjects compared with controls, but no significant differences in plasma levels of IL-6 and TNF-α were observed [5]. Thus, the current findings may indicate common reduction of IL-1β across OCD and trichotillomania, but that trichotillomania is linked with a broader range of inflammatory changes.

Several limitations need to be considered in relation to the current study. The study did not include a control group, and so analyses were correlational in nature, and used external published norms as a reference. We did not record the duration of illness. As noted, we used saliva samples, which may only weakly reflect central inflammatory pathways. This being an exploratory study, we did not correct for multiple comparisons.

In summary, relatively low level of salivary inflammatory cytokines were observed in people with trichotillomania. Follow-up work is now needed to examine inflammation using blood measures in people with this condition, ideally also including a normative control group (not included in the current design). If blood measures show the same pattern of results as seen here with saliva, trichotillomania may be distinct from other mental disorders (such as OCD, schizophrenia, and depression), which have been associated tentatively with increased rather than decreased peripheral inflammation [4]. Inflammatory findings in mental disorders, however, are highly heterogeneous and may reflect distinct subtypes.

## Funding and Disclosure Statement

This research was supported by a research grant from the TLC Foundation for Body Focused Repetitive Behaviors to Dr. Jon E. Grant and a Wellcome Trust Clinical Fellowship to Dr. Samuel R. Chamberlain (110049/Z/15/Z). Dr. Jon E. Grant has received research grants from the American Foundation for Suicide Prevention, NIAAA, and Takeda Pharmaceuticals. Dr. Jon E. Grant receives yearly compensation from Springer Publishing for acting as Editor-in-Chief of the Journal of Gambling Studies and has received royalties from Oxford University Press, American Psychiatric Publishing, Inc., Norton Press, and McGraw Hill. Dr. Samuel R. Chamberlain consults for Cambridge Cognition and Shire. There are no competing financial interests in relation to this work.
